# Wild boar (*Sus scrofa*) increases species diversity of semidry grassland: Field experiment with simulated soil disturbances

**DOI:** 10.1002/ece3.4950

**Published:** 2019-02-05

**Authors:** Eva Horčičková, Josef Brůna, Jaroslav Vojta

**Affiliations:** ^1^ Faculty of Science, Department of Botany Charles University in Prague Prague Czech Republic; ^2^ Faculty of Science, Institute for Environmental Studies Charles University in Prague Prague Czech Republic; ^3^ Institute of Botany The Czech Academy of Sciences Průhonice Czech Republic

**Keywords:** abandoned landscape, *Brachypodium pinnatum*, disturbance, experiment, semidry grassland, species diversity, *Sus scrofa*, *Thymus pulegioides*, wild boar

## Abstract

**Background:**

Foraging activities of wild boar (*Sus scrofa*) create small‐scale soil disturbances in many different vegetation types. Rooting alters species composition by opening niches for less‐competitive plants and, as a recurrent factor, becomes a part of the community disturbance regime. Vegetation responses to wild boar disturbance have mostly been studied in the boar's non‐native range or in native forest, rather than in open habitats in the native range. We investigate the response of open European semidry grassland vegetation dominated by *Brachypodium pinnatum* to native wild boar pressure in an abandoned agricultural landscape.

**Methods:**

To describe the disturbance regime, we repeatedly mapped rooted patches during a 5‐year period. Additionally, to study the vegetation response, we performed an artificial disturbance experiment by creating 30 pairs of simulated disturbances and undisturbed plots. The vegetation composition of the paired plots was repeatedly sampled five times in eight years of the study.

**Results:**

Based on repeated mapping of disturbances, we predict that if the disturbance regime we observed during the 5‐year period were maintained over the long term, it would yield a stable vegetation ratio consisting of 98.7% of the grassland undisturbed, 0.4% with fresh disturbance, and 0.9% in older successional stages.

Vegetation composition in the artificially disturbed plots was continuously converging to that of undisturbed vegetation, but these disturbed plots still differed significantly in composition and had higher species number, even after eight years of succession.

**Synthesis:**

Our results thus show that wild boar disturbance regime in its native range increases heterogeneity and species diversity of semidry grassland vegetation.

## 1 INTRODUCTION

Disturbance structures natural communities by opening regeneration niches for less‐competitive species (Sousa, 1984). Parameters such as the frequency and severity of disturbance define the disturbance regime, which is a major determinant of long‐term species composition, diversity, and physiognomy of the community (Hobbs & Huenneke 1992; Shea, Roxburgh & Rauschert, (2004). European temperate‐zone open semidry grasslands are strongly dependent on a disturbance regime. Most of them were historically formed and maintained by both wild ungulates and management activities including the pasturing of domestic animals (Bornkamm, 2006). Since the last decade of the twentieth century, changes in European land use policy have been leading to agricultural abandonment of large areas (Coffin, Lauenroth, & Burke, 1996; Cremene et al., 2005), which has reduced the impact of domestic animals and increased the importance of wild ungulates in landscape dynamics.

Among European wild animals, wild boars (*Sus scrofa*) provide a unique type of disturbance regime. Although they do also graze, wild boars are better known for altering vegetation types by disturbing the soil (Ballari & Barrios‐García, 2014). Their rooting disrupts and overturns vegetation usually along with the topsoil to a depth of 5–15 cm, creating disturbances in areas ranging from a few square centimeters to thousands of square meters. The areas disturbed vary annually, seasonally, and among habitat types (Welander, 2000). Variation in wild boar rooting intensity yields varying effects on such soil properties as nutrient availability, moisture (Bueno, Azorín, Gómez‐García, Alados, & Badía, 2013; Kotanen, 1997; Mohr, Cohnstaedt, & Topp, 2005; Tierney & Cushman, 2006), bacterial community structure (Wirthner, Frey, Busse, Schütz, & Risch, 2011), and seed‐bank species richness (Bueno, Reiné, Alados, & Gómez‐García, 2011).

Although the wild boar is originally a Eurasian species, it has been introduced and, due to its large ecological amplitude, successfully established in a broad range of habitats on other continents (Barrios‐Garcia & Ballari, 2012). There is abundant documentation of vegetation responses to rooting in regions hosting non‐native wild boar or feral pig populations. Free‐ranging boars are thus seen as an economic risk to agricultural systems (Bankovich, Boughton, Boughton, Avery, & Wisely, 2016; Campbell & Long, 2009) as well as a threat to diversity in valuable natural communities (Felix, Orzell, Tillman, Engeman, & Avery, 2014). Especially on islands with no historic native ungulate fauna, introduction of wild boars has been shown to cause decline of native species and support plant invasions (Aplet, Anderson, & Stone, 1991; Oldfield & Evans, 2016); however, there are also examples of boar rooting that has not resulted in negative vegetation responses (Baron, 1982).

In the native range of wild boars, studies on their effects have been motivated in large part by their continuous increase in population size and the challenges this poses to economic interests (Massei et al., 2015) in agriculture (Bobek, Furtek, Bobek, Merta, & Wojciuch‐Ploskonka, 2017) and forestry (Gómez & Hódar, 2008; Groot Bruinderink & Hazebroek, 1996). Examination of the responses of vegetation composition to wild boar rooting in their native range has been focused mostly on forest understory. Studies have shown both increases and decreases in diversity (Brunet, Hedwall, Holmstr, & Wahlgren, 2016), depending on disturbance intensity (Burrascano et al., 2015). In comparison with forests, investigation of wild boar disturbances in open habitats in their native distribution has been scant (Barrios‐Garcia & Ballari, 2012). This includes an experiment conducted in semidry grassland vegetation using both artificial and natural disturbances, which showed changes in plant species and trait composition. Artificial soil disturbances had greater species richness than undisturbed grassland and, together with natural disturbances, were characterized by protohemicryptophytes and in general smaller species with no or moderate seed dormancy. However, that study lasted only five months, so described only beginning of succession (Lavorel, Touzard, Lebreton, & Clément, 1998). Despite the increasing conservation importance of understanding European open grassland dynamics, long‐term studies about wild ungulate disturbances in this community type are still missing.

The purpose of the present study is to assess the extent and dynamics of wild boar rooting in an open semidry grassland and discern how these affect vegetation characteristics. We hypothesize that rooting by wild boar can alter semidry grassland composition by supporting less‐competitive species. Specifically, we aim to (a) quantify the natural frequency of wild boar disturbance; (b) document changes in species composition and their development over time; (c) directly assess the relationships of alpha and beta diversity to disturbance.

For these purposes, we chose open semidry vegetation dominated by the competitive grass *Brachypodium pinnatum*. In this vegetation type, we recorded patches of natural wild boar disturbances during five consecutive field seasons. We also established an eight‐year‐long field experiment to study succession on patches where we simulated wild boar rooting and compared vegetation on these artificial disturbances to vegetation on undisturbed plots.

## 2 METHODS

### 2.1 Study area

The investigation was carried out in Military Area Hradiště in the Doupov Mountains, western Czech Republic (50°18′25″N, 13°04′50″E; 502–736 m a.s.l.). The mean annual temperature is approximately 6°C, and the mean annual precipitation is approximately 670 mm (Vesecký et al. 1961).

Our study site is part of the safety buffer zone around the active military training ground. The previously cultivated landscape has been abandoned since 1953 and has thus been free of agricultural and touristic pressure. Secondary succession has led to a mosaic of shrubs and forests. Our study is focused on remnants of semidry grasslands that occupy small open enclaves on slopes with approximately southern orientations. The focal grassland can be classified mostly as tall‐herb vegetation of the *Festuco‐Brometea* class dominated by the grass *B. pinnatum*, but it also contains patches of drier short‐herb vegetation closer to the class *Sedo‐Scleranthetea* (Braun‐Blanquet 1955). Wild boar (*S. scrofa*), red deer (*Cervus elaphus*), and non‐native sika deer (*Cervus nippon*) are common wild ungulate species in the area. Fresh rooting by wild boar is clearly distinguishable from other soil disturbances like erosion or burrows of foxes and badgers, as well as from smaller disturbances made by rodents.

### 2.2 Study design

**Figure 1:**
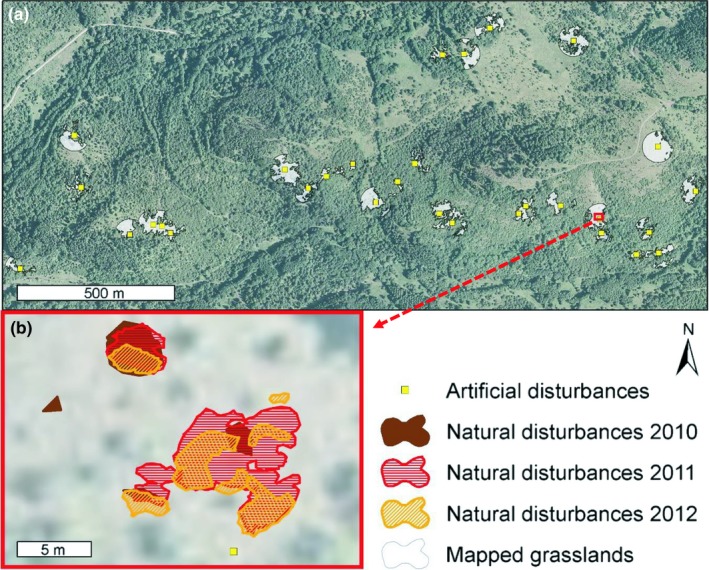
(a) Distributions of mapped open grassland (white semitransparent patches) and artificial disturbances used in the experiment (yellow squares); (b) Detail of mapped fresh wild boar disturbances—an example of a disturbed patch partly re‐rooted in three

The selection of particular open grasslands enclaves for the study was based on several earlier vegetation analyses of grasslands in the respective area (Vojta unpublished data). A raster digital elevation model was used to choose areas with environmental conditions represented by topographic wetness index values (SAGA Development Team 2007) in the range 4–7, which was extracted from already‐known areas of the target vegetation type. This gave us polygons which could potentially host the species‐rich grassland vegetation. Unsuitable objects features such as paths, forests, and bare rocks were excluded from the selected polygons manually so that their areas would not be included in the measured areas of the polygon. The total potentially useful area was reduced to 30 circles of 50 m radius around randomly selected points, minus the unsuitable objects. The final area of studied vegetation was 71 688 m^2 ^(Figure 1), with the selection made in ArcGIS (ESRI, 2016).

#### 2.2.1 Field mapping of natural disturbances

To quantify the natural frequency of wild boar disturbance, occurrence of all visible natural disturbances was mapped in the selected area. The enclaves were searched each spring and autumn in the years 2008–2012, and disturbances were delineated using a differential GPS. GPS data were postprocessed in Trimble Pathfinder Office using CZEPOS correction data resulting in approximately <0.5 m precision. Polygons of rooted soil were classified as fresh disturbances characterized by no vegetation cover or as particular older stages based on their approximated age. To unify and verify the information about disturbance age, a layer of previously mapped polygons was used for comparison. No rooted patch had an area larger than that of a 50‐m circle. If the disturbance was only partly in the circle, we mapped its entire area for disturbance size distribution data, but used only the part in the circle for calculation of rooting probabilities. The final classification and analysis of patch areas and their overlaps in time were done in ArcGIS (ESRI, 2016).

#### 2.2.2 Field experiment using artificial disturbances

To document changes in species composition and their development over time, artificial soil disturbances were created in autumn 2007. Thirty pairs of 0.5 × 0.5 m permanent plots were designated at random in the same area of open grasslands as the field mapping. The placement of permanent plots was based on randomly selected points, but each was corrected to be located in the closest patch of homogenous tall‐grass vegetation without signs of previous disturbance (Figure 1).

Plant species composition on permanent plots was estimated visually using a percentage scale for cover of all vascular plant species. One plot in each pair was disturbed by removing the vegetation along with the top 20 cm of soil, which imitated actual wild boar disturbances observed in the same area. The size of the disturbed square was 1 m^2^, so it is larger than the sampled plot and the sampled plot is in the centre of disturbance to avoid an edge effect (Supporting Information Figure S1). The second square was left undisturbed as a control plot. The distance between paired squares was 0.5 m. The 1 m^2^ size of the disturbance is a common disturbance size according to the literature (Felix et al., 2014; Kotanen, 1995; Welander, 2000), and this method had been already used for simulation of wild boar rooting in a semidry grasslands (Lavorel et al., 1998).

Vegetation recording on both disturbed and control permanent plots was done annually using the same technique each spring of the years 2008–2012. It was also done on 27 of these pairs of plots in spring 2015, with three plots having been destroyed by wild boar after the fifth year of the experiment, rendering those pairs unusable. Vegetation data from only seven control plots were collected in 2007, as were data from all 30 of the experimentally disturbed plots, prior to their artificial disturbance.

The simulation gave us an advantage of knowing previous vegetation, possibility to choose disturbed and control plots of the same history and limit other factors such as size of the disturbance, which plays an important role in succession (Bullock, Hill, Silvertown, & Sutton, 1995). On the other hand, experiment ignores many factors existing in the real system (e.g., animal preferences or potential removal of eaten matter), so we also sampled available natural disturbances caused by wild boar.

#### 2.2.3 Comparison of artificial and natural disturbances

Between 2007 and 2011, several natural disturbances of similar sizes to our artificial disturbances were permanently marked along with nearby undisturbed vegetation. In 2011–2013, those pairs of plots in which neither plot had been destroyed by wild boars were sampled with the same method as plots in our field experiment. This gave us an additional dataset for 45 pairs of natural disturbances of ages ranging from 1 to 6 years and accompanying undisturbed plots that were not recorded as rooted during any of our field mapping (Supporting Information Table S1).

In order to enable comparison between natural and artificial plots, we also used data from 45 paired plots from our manipulation experiment. The subset was chosen to be as similar as possible to the natural disturbance dataset in two parameters—time and location. For each pair of natural plots, we chose corresponding artificial pair from the same enclave and of the same age, if possible. In cases in which there were more natural disturbances of the same age on the same enclave, we would also include an artificial plot of the same age from the spatially closest enclave. We did not have 6‐year‐old plots in our experimental dataset, so we used 8‐year‐old plots instead (Figure 1).

### 2.3 Data analysis

#### 2.3.1 Field mapping of natural disturbances

Total rooted areas per year, polygon size distribution, and all combinations of rooting history (e.g., rooted in years 1 and 2 or rooted only in year 3) were analyzed in ArcGIS (ESRI, 2016). Because the total rooted areas and overlaps of stages differed between years, this resulted in a database in which every part of each disturbance has its own history throughout the study period (e.g., if half of a disturbed patch was re‐rooted in a given year, then the two parts would have different disturbance histories). We used these field data to create a projection matrix (Supporting Information Table S3) of a visible disturbance existence cycle (Figure 3) using the function *stable.stage* from the *popbio 2.4.3 *package in R (Stubben & Milligan 2007). Maximum disturbance existence time was defined as four years, because it was very difficult to find rooted patch after this amount of time had elapsed. Transition probabilities of disturbance stages were averaged from all observations of the specific transition. Randomness of rooting overlaps in time was tested by chi‐square test, using *chisq.test* *R function* (R Development Core Team, 2011), comparing the expected probability of being rooted based on the grassland–disturbance ratio at the particular time to the observed overlaps.

#### 2.3.2 Field experiment using artificial disturbances

Vegetation composition was analyzed by multivariate methods. Logarithmic transformation was used for vegetation data. Principal components analysis (PCA) was used to quantify maximal variability explained by alternatively one or four unconstrained axes. Difference between control plots and one‐year‐old disturbances was tested by partial redundancy analysis (RDA), where enclave (a spatial parameter) was used as a covariate. Interaction between time and treatment was tested by RDA with permutation testing in split plot design. Principal response curves (PRC) were used to describe the main change in species composition. Principal coordinates analysis (PCO) was used for visualization of time–treatment group relative positions. Multivariate methods were mostly performed by CANOCO 5 software (Ter Braak & Šmilauer, 2012).

Species richness (number of species per sample) on artificial disturbances and control plots was compared by negative binomial generalized linear model with log link function using *glm.nb* function in MASS package version 7.3‐45 (Kafadar, Koehler, Venables, & Ripley, 1999) and tested by ANOVA function with chi‐square test. Beta diversity was used to characterize the difference in time–treatment group homogeneity and was calculated in terms of Bray–Curtis distances in principal coordinates analysis (PCO) ordination space using the *betadisper* function from Vegan R package (Oksanen et al., 2017).

#### 2.3.3 Comparison of artificial and natural disturbances

To assess how well the experimental manipulation was representative of actual wild boar disturbance, the vegetation on artificial and natural disturbances was compared by partial canonical correspondence analysis (CCA). Logarithmic transformation was used for response data. Enclave (a spatial parameter), age of disturbance, year of data collecting, and identity of permanent experimental plot were used as covariates.

## 3 RESULTS

### 3.1 Field mapping of natural disturbances

**Figure 2:**
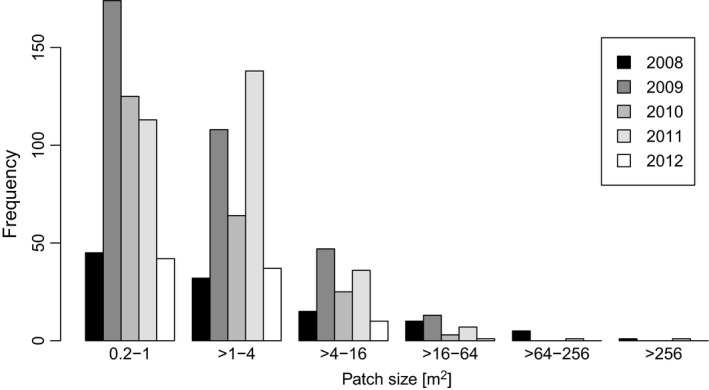
Size distribution of fresh natural disturbances caused by wild boar rooting in 5 years

Rooted patch sizes varied between 0.2 and 312 m^2^ with a median of 1 m^2 ^and mean of 4 m^2^ (Figure 2). Total areas of fresh disturbances differed among years. Of 71 688 m^2^ of semidry grassland studied, the freshly rooted area per year ranged from 153 to 516 m^2^. Freshly disturbed area had significantly (ANOVA, *p* = 0.01, *F* = 14.64) greater extent during spring (mean 312 m^2^) than in autumn (mean 29 m^2^). Between 3% and 30% of the fresh disturbances appeared on already rooted patches. Rooting occurrence was not spatially random (chi‐square, *X* ^2 ^= 1949.1, *p* < 0.0001). During the five years of field mapping, several patches were rooted more than twice (three or four times). We recorded 30 combinations of years in which rooting was done, but there was no plot freshly rooted every year, that is, five times (Figure 2)

**Figure 3:**
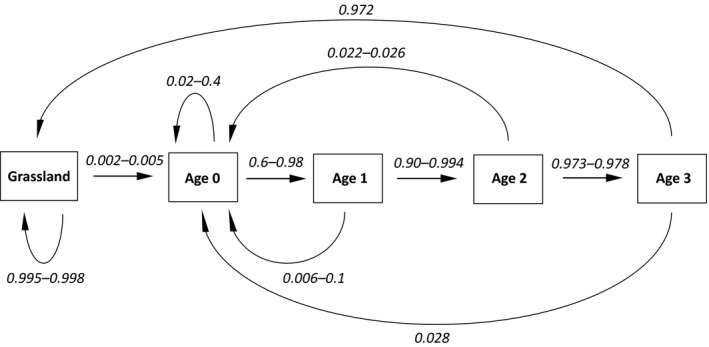
Graph of five‐stage existence cycle of natural disturbance in grassland. These stages comprise undisturbed grassland (grassland), fresh disturbance with bare soil (age 0), disturbances 1–3 years old (ages 1–3, respectively). One step is one year. Arrows show nonzero probabilities of transition, with numbers indicating transition probabilities. In contrast to transitions from age 3, transitions from younger stages were observed more frequently, yielding ranges of transition probabilities

The stable stage distribution of the disturbance regime based on five years of repeated mapping is 98.68% grassland, 0.38% fresh disturbances, 0.33% one‐year‐old patches, 0.31% two‐year‐old patches, and 0.3% three‐year‐old patches (Figure 3).

### 3.2 Field experiment using artificial disturbances

Species composition on artificial disturbances differed strongly from control plots the first year after disturbance (partial RDA, pseudo‐*F* = 12.3, *p* = 0.002, first axis explained 30% of data variability and 33% of data variability was explained by the first axis in PCA). Species with the best fit to the first axis were *Arabidopsis thaliana*, *Viola arvensis, Hypericum perforatum, Euphorbia cyparissias, *and *Rumex acetosella *on the side of disturbances and* B. pinnatum, Fragaria viridis, Achillea millefolium, Festuca rubra, *and* Arrhenatherum elatius *on the side of control plots (Supporting Information Table S4). The treatment–time interaction was significant for every year of data collecting after disturbance (Supporting Information Table S2) and even eight years after disturbance (RDA, pseudo‐*F* = 6.8, *p* = 0.002; 2.74% of data variation was explained by first axis in RDA and 7.44 by first axis in PCA).

Species with the strongest responses to the interaction between treatment and time eight years after disturbance were *Thymus pulegioides, H. perforatum, *and *Potentilla argentea*, followed by *Trifolium repens, E. cyparissias, Lotus corniculatus, Trifolium arvense,* and *R. acetosella* in showing the biggest increases over time on disturbed plots. Control plots were represented by *B. pinnatum* at the first place and then *A. millefolium* and *F. viridis*. These three species were stable in leading the interaction on control plots in all years of the experiment (Supporting Information Table S2).

**Figure 4:**
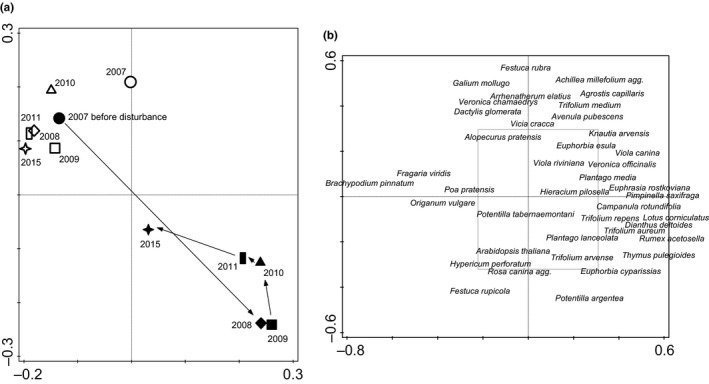
Ordination of vegetation composition from the field experiment. (a) Plotted are centroids of treatment–time groups in principal coordinates analysis (PCO) projection using Bray–Curtis distances. Open symbols represent results from control plots, filled symbols represent results from disturbed plots and from 30 plots before disturbance in 2007. Arrows show the direction of vegetation change on disturbed plots over time. First two axes explain 19.7% of the data variability. (b) The same ordination space with plotted 40 most frequent species. Grey rectangular shows the position of (a) part of the picture

The differences between treatment and time groups is shown using PCO with the first two axes explaining 19.7% of the data variability (Figure 4).

**Figure 5:**
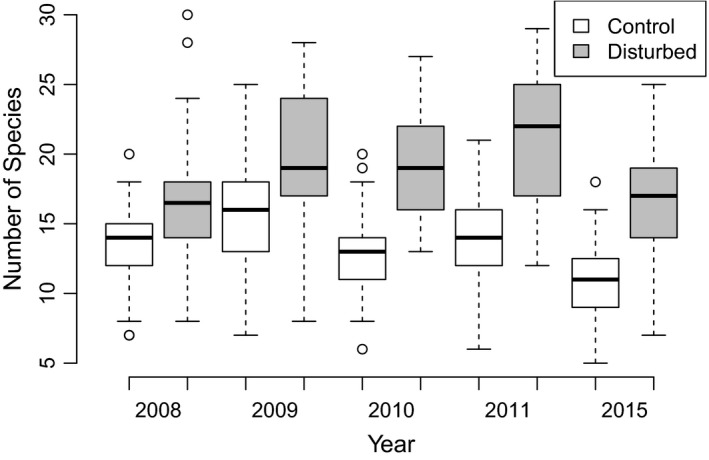
Number of vascular plant species on artificial disturbances and control undisturbed plots in treatment–time groups. For years 2008–2011, *n* = 30 per treatment; for 2015, *n* = 27

Species richness showed significant responses to treatment, and interaction between treatment and time (Figure 5, Table 1). The species richness was significantly higher on disturbed plots in all years after disturbance (in 2008, *p* = 0.000644; in 2009, *p* = 0.000219; in 2010, *p* < 0.0001; in 2011, *p* < 0.0001; and in 2015, *p* < 0.0001). The number of species on disturbed plots varied between 7 and 30 (18.74 ± 4.9, mean ± *SD*) and on undisturbed control plots between 5 and 25 (13.37 ± 3.68, mean ± *SD*). The highest numbers of species were observed on several disturbed plots in the fourth year after rooting. Eight years after disturbance, the mean number of species on disturbed plots was 16.67 (±4.52 *SD*) and on control plots 10.67 (±3.13 *SD*) (Figure 5; Table 1).

**Table 1:** Test of the effect of treatment (disturbance × undisturbed control) and time on the species richness

	*df*	Deviance	Resid. *df*	Resid. Dev	*p*
NULL			293	422.51	
Treatment	1	118.322	292	304.19	<0.0001
Time	1	2.245	291	301.94	0.134
Treatment:time	1	7.239	290	294.71	0.007

**Figure 6:**
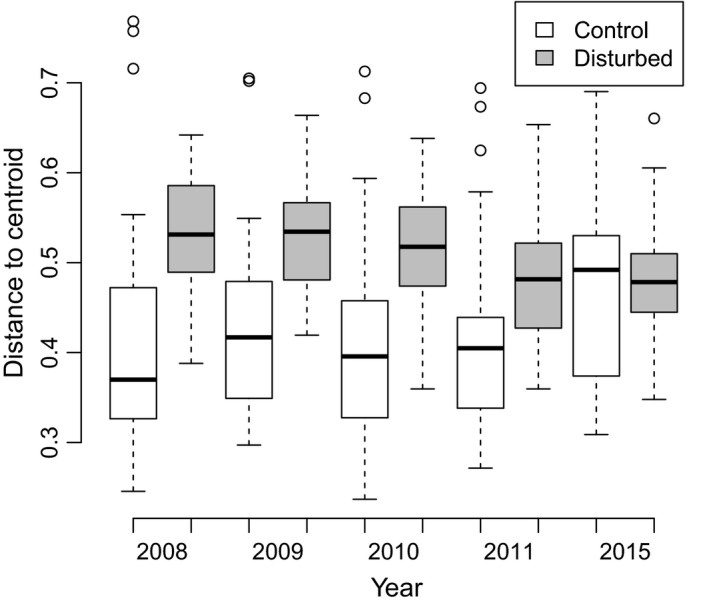
Beta diversity of vascular plant species on artificial disturbances and control plots expressed in terms of multivariate dispersion in principal components analysis (PCA) within treatment–time groups

Vegetation composition on artificial disturbances differed strongly between plots during the first three years after digging. This is shown by the beta diversity being significantly higher than on control plots in 2008–2010 (Tukey's HSD in 2008, *p* < 0.0001; in 2009 and 2010, *p* = 0.001). The vegetation composition was continuously converging over time, and beta diversity was no longer significantly different from control plots by 2011 (Tukey's HSD *p* = 0.38). Also, there was not a significant difference in beta diversity between control plots in 2015 and disturbed plots in 2011 (Figure 6). The whole ANOVA model was significant (*F* = 8.24, *p* < 0.01). There was no significant difference in beta diversity between the groups (future disturbances and controls) in 2007 and between these plots in 2007 and control plots in 2008. Differences between time groups of disturbed plots are not significant in any combination (grey boxplots, Figure 6).

### 3.3 Comparison of artificial and natural disturbances

We compared the vegetation from our experiment with the smaller dataset of natural plots (Supporting Information Figure S2). Experimental and natural plots differed significantly (partial CCA, pseudo‐*F* = 2.5, *p* = 0.002; the difference explained 1.2% of variability). Treatment (disturbance vs. control/undisturbed plot) was also significant (partial CCA, pseudo‐*F* = 10.6, *p* = 0.002; the difference explained 5.4% of variability).

## 4 DISCUSSION

### 4.1 Field mapping of natural disturbances

This paper examined soil disturbances created by native wild boar (*S. scrofa*) in central European semidry grassland (dominated by *B. pinnatum*). Each year, about 0.2% to 0.7% of the area of studied vegetation was rooted during our 5‐year‐long field mapping. It is approximately half in comparison with wild boar rooting in Swedish humid grasslands (Welander, 2000). And it is in general lower than the percentage of rooted ground documented in other studies from various habitats. The relatively low rate we found could be due to the fact that rooting is usually lower in dry conditions. For instance, the percentage of rooting in an area in Spain with a dense wild boar population and a Mediterranean climate was 0.23 (Cahill, Llimona, & Gràcia, 2003), thus similar to or lower than in our study site.

Up to 30% of the fresh disturbances we observed were re‐rooted the following year. Repeated rooting might keep the vegetation on disturbances in earlier successional stages for a longer time, while other patches would stay untouched. A partly repeated pattern of disturbance pressure is known to increase spatial heterogeneity, as has been shown for example in a study of horse grazing (Adler, Raff, & Lauenroth, 2001). Partial re‐rooting by feral pigs in two or three seasons has been described from a mosaic landscape of wet pineland savannas in Florida (Felix et al., 2014). In our system, several patches were rooted three or even four times, but none were rooted every year of the observation. So, areas with soil that is continuously bare, due to rooting, for five or more years would not appear very likely. We expect that if the frequency of disturbances would follow the same dynamics as during our 5‐year‐long observation, the system would stably maintain proportions of 0.38% freshly disturbed soil and 0.94% older, but still visible, successional stages.

The total disturbed area recorded during spring was ten times higher than in autumn. In two seasons, we repeated the mapping also in summer, but in each season, <1 m^2^ of fresh disturbance was found. This means that most of the disturbances were created in winter and spring. Other studies have also reported more intense rooting during the cold part of the year (Dovrat, Perevolotsky, & Ne’eman, 2014; Welander, 2000). The reason might be lower availability of aboveground food.

The size distribution of observed rooted patches was highly skewed, with the median 1 m^2^ and the mean 4 m^2^. Other studies have reported similar distributions, but shifted slightly to smaller patches, with most of the disturbances sizes around 1 m^2^ (Felix et al., 2014; Kotanen, 1995; Welander, 2000). The other studies usually do not mention any bottom threshold for patch recording and report even very small disturbances, for example, 0.0023 m^2 ^(Felix et al., 2014). We did not map disturbances smaller than 0.2 m^2^, because it was not possible to check whether such a small patch of bare soil was created by wild boar. This decision and the existence of one very large disturbance might have shifted the mean size.

### 4.2 Field experiment using artificial disturbances

Eight years after our artificial rooting, the vegetation on disturbed plots still differed from undisturbed vegetation in terms of composition and species number (higher on the disturbed plots). Undisturbed vegetation was stable in time, with a mean of 13 species per plot and was mostly dominated by *B. pinnatum*. On disturbances, in contrast, the main dominant became *T. pulegioides*, and the maximum number of species reached 30. For comparison, the most species recorded from the same size sample in open grasslands is about 45, observed in the Czech Republic (Chytrý et al., 2015). One year after rooting, disturbed plots were characterized by several annual ruderals such as *A. thaliana* and *V. arvensis* or stress‐tolerant ruderal *Myosotis stricta*. There were also several C‐S‐R strategists, for example, *E. cyparissias*, *R. acetosella,* or *Trifolium arvense* and even a competitor *H. perforatum*. In following years was increasing proportion of C‐S‐R strategists. The results of the experiment supported our hypothesis that rooting can alter grassland composition by enhancing less‐competitive species, and in the case of our study system, it increased species diversity. This result is in sharp contrast with most studies on wild boar and feral pigs, which have reported diversity losses (Aplet et al., 1991; Bankovich et al., 2016; Campbell & Long, 2009; Kotanen, 1997). However, the cited studies are from regions where wild boar is not a native species, and in most of them, the authors aimed to uncover the potential threat to vegetation diversity. There are other factors, for example, spatial and temporal scales, making results less comparable. One of them is a size of sampled plots, which varied between cited studies from 0.125 m^2^ (Kotanen, 1997) to 50 m^2^ (Aplet et al., 1991). Effects of disturbance on species diversity are known to vary with spatial scale and might even give contradictory results. For example, a study of cattle grazing in Argentinian pampa shown that herbivory increased diversity on small plots, but reduced it at scales larger than 120 m^2^ (Chaneton & Facelli, 1991). Our experimental disturbances belong to small plots in comparison with other mentioned studies, so the experiment is more likely to show increase of diversity. On the other hand, one of the studies reporting decrease of diversity was based on even smaller plots (Kotanen, 1997). Apart from number of species, disturbance also increased beta diversity. Vegetation response to rooting differs strongly between plots mostly first year after the disturbance. In following two years, the response was slowly converging and successional paths were not significantly different between plots fourth year after the disturbance. Shortly after disturbance, there might be more stochastic processes influencing species composition, but after four years, there are mostly the same species profiting from disturbance.

Vegetation composition on our artificial plots was in general converging over time and returning to the stage of undisturbed vegetation, but the succession process takes more than eight years to finish (Figure 4). This might be caused mainly by slow return of the original dominant species—*B. pinnatum*. In large disturbances, this vegetation type can react very slowly, even taking decades (Bornkamm, 2006; Coffin et al., 1996). However, for smaller disturbances such as wild boar rooting such a slow response is not typical (Baron, 1982). If the vegetation has an evolutionary history of being subjected to such disturbance, the composition returns to the original stage in one or two years (Dovrat et al., 2014). In case of our study area, the vegetation has a long evolutionary history in common with wild boar. In the decades before abandonment, presence of wild ungulates was strongly regulated; however, the area was mostly used as extensive pastures. The plant community might need similar evolutional adaptation for both extensive management and disturbance regime provided by wild ungulates. So, the slow return of the vegetation before our experimental disturbance in surprising.

### 4.3 Comparison of artificial and natural disturbances

The behavior of naturally disturbed patches, as well as their corresponding undisturbed plots, differed significantly from experimental data. This might have been caused by several factors. The experimental plots were chosen for their vegetation composition and also specifically for absence of disturbance. The locations of natural disturbances were chosen by wild boar, probably for foraging purposes. We know when these disturbances appeared and that the corresponding undisturbed plots had not been disturbed in the several previous years. However, from our experimental data, it is obvious that the effect of previous rooting on vegetation is even longer. This might be the main explanation for the high heterogeneity of these undisturbed plots. Another potential factor was the timing of rooting. All our artificial disturbances were created during one week in autumn 2007. In contrast, the natural disturbances were created at various times, mostly during winter. The spatial distribution of natural disturbances could also have influenced our results, as they were obviously grouped in time and space. During the field mapping, we observed natural disturbances in all grassland enclaves, but some of them were unsuitable for sampling because they were the wrong size or there was an absence of nearby undisturbed vegetation or because re‐rooting occurred during the study. These complications show the challenges of relying upon an un‐manipulated natural system to provide the data.

The ability of the experiment to capture the effects of disturbances is shown by the fact that the overall differences between all disturbances and all undisturbed plots were stronger than the differences between natural and experimental plots. Indeed, the main pattern in differences between disturbances and undisturbed vegetation was the same for both natural and experimental plots (Supporting Information Figure S2). Species composition of naturally and artificially disturbed sites of the same age was very similar. One difference, however, was the higher proportion of *B. pinnatum* on experimental plots—probably because of their already‐mentioned different histories. There were only two species found exclusively on natural disturbances: *Convolvulus arvensis on *several plots and *Calamagrostis epigejos* on one plot.

Wild boar rooting has often been linked with promotion of invasive plants (Bankovich et al., 2016; Oldfield & Evans, 2016). Our study did not directly address this topic. Although at least one common invasive—*Lupinus polyphyllus*—and one expansive (i.e., native, but colonizing new, anthropogenic habitats (Pyšek et al., 2004)) plant species—*C. epigejos*—occur frequently in the study area, experimental plots were not originally placed in vegetation containing these species. Nevertheless, both plants were found in mapped polygons around the experimental plots. Neither of them appeared in any experimental plot during our study, and there was only one record of *C. epigejos* on a natural disturbance, but we do not know whether this species was there before rooting.

In summary, the results of this study show that rooting of native wild boar (*S. scrofa*) in semidry grasslands dominated by *B. pinnatum* increases heterogeneity and species richness of the vegetation. The effect lasts for more than eight years and continues to influence the vegetation composition even after the disturbance is no longer visible.

## CONFLICT OF INTEREST

None declared.

## AUTHORS’ CONTRIBUTION

JV and EH provided idea and designed the study; EH acquired and interpreted the data and drafted the article; JB analyzed the spatial data (GIS); EH and JV analyzed vegetation data; JB and JV revised the text; and EH, JB, and JV finally approved the version to be submitted.

## Data Availability

Data available from the Dryad Digital Repository: https://doi.org/10.5061/dryad.4521vq2. Data files: Experimental_disturbances_species, Natural_disturbances_species, Rooted_area.
