# Supplementary figures and images for: Wild boar (Sus scrofa) increases species diversity of semidry grassland: Field experiment with simulated soil disturbances
Source: Ecol Evol. 2019 Feb 5;9(5):2765–74. doi: 10.1002/ece3.4950 (PMC6405487; doi:10.1002/ece3.4950)

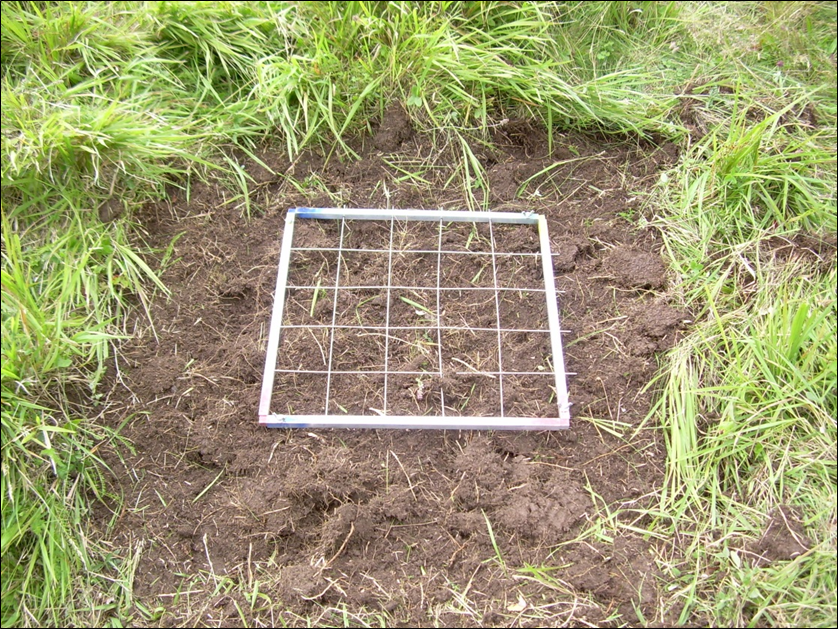

Supplement: Supplementary file 1 [file ECE3-9-2765-s001.png]

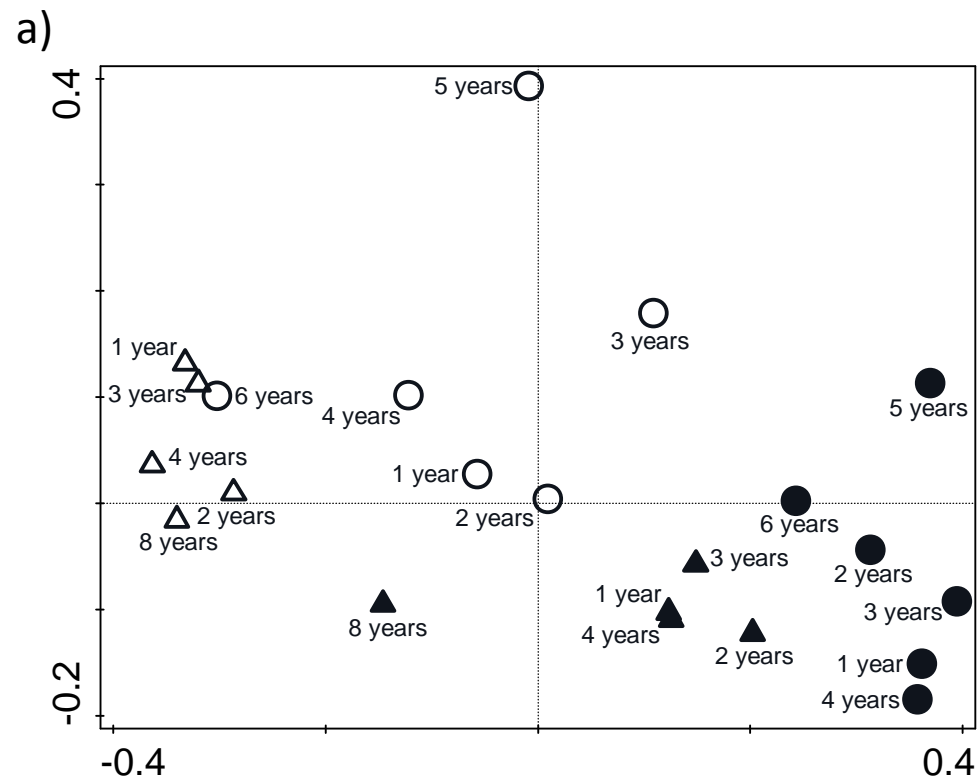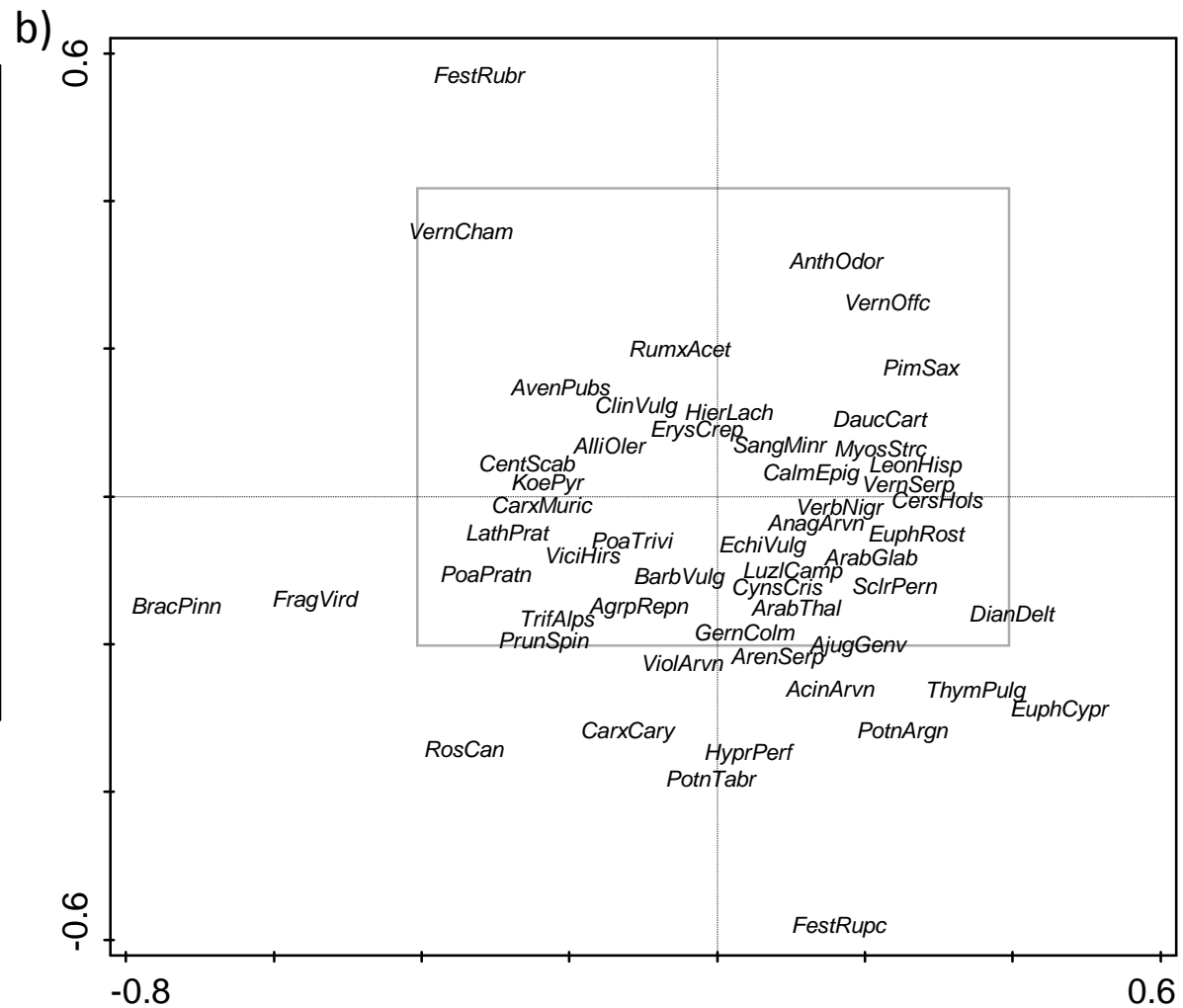

Supplement: Supplementary file 2 [file ECE3-9-2765-s002.pdf]
