# Supplementary material for: Wild boar (Sus scrofa) increases species diversity of semidry grassland: Field experiment with simulated soil disturbances
Source: Ecol Evol. 2019 Feb 5;9(5):2765–74. doi: 10.1002/ece3.4950 (PMC6405487; doi:10.1002/ece3.4950)
Supplement: Supplementary file 3 [file ECE3-9-2765-s003.docx]

(Fig.S1)

**Table S1** Number of pared (rooted and unrooted) vegetation samples from naturel disturbances caused by wild boar.

| Age (years) | n (pairs) | Location (polygon number) |
| --- | --- | --- |
| 1 | 7 | 2, 10, 11, 18, 18, 20, 20 |
| 2 | 7 | 2, 10, 11, 18, 20, 21, 28 |
| 3 | 5 | 18, 20, 23, 27, 28 |
| 4 | 14 | 4, 4, 11, 11, 18, 18, 21, 23, 27, 27, 25, 25, 30, 30 |
| 5 | 7 | 3, 11, 21, 21, 21, 27, 25 |
| 6 | 5 | 3, 4, 14, 21, 29 |

(Fig.S2)

Experimental and natural plots differ significantly (Partial CCA, pseudo-F=2.5, P=0.002; the difference explained 1.2 % of variability). Treatment (disturbance or control plot) was also significant (Partial CCA, pseudo-F=10.6, P=0.002; the difference explained 5.4 % of variability)

**Table S2** Redundancy Analysis of treatment-time interaction in particular time periods of the field experiment. Last two columns contain list of plant species with the strongest response to the interaction.

| Time period | N of pairs | Explained variation  RDA (%) | Adjusted expl. var. RDA (%) | Pseudo-F | P | 4 axes  RDA (%) | Explained variation  PCA (%) | 4 axes  PCA (%) | Partial variation | Strongest response to the treatment-time interaction | |
| --- | --- | --- | --- | --- | --- | --- | --- | --- | --- | --- | --- |
|  |  |  |  |  |  |  |  |  |  | Disturbances | Undisturbed plots |
| 2008-2009 | 30 | 1.8 | 0.6 | 1.6 | 0.002 | 21.63 | 7 | 25.62 | 1138.073 | *Hypericum perforatum*  *Trifolium repens*  *Potentilla argentea*  *Trifolium arvense*  *Rumex acetosella*  *Euphorbia cyparissias* | *Brachypodium pinnatum*  *Fragaria viridis*  *Achillea milefolium*  *Festuca rubra* |
| 2009-2010 | 30 | 1.5 | 0.3 | 1.3 | 0.002 | 21.5 | 7.23 | 25.58 | 1253.328 | *Potentilla argentea*  *Thymus pulegioides*  *Hypericum perforatum*  *Trifolium repens*  *Lotus cornuculatus*  *Euphorbia cyparissias* | *Brachypodium pinnatum*  *Achillea milefolium*  *Fragaria viridis*  *Dactylis glomerata*  *Galium mollugo*  *Festca rubra* |
| 2010-2011 | 30 | 1.2 | <0.1 | 1 | 0.002 |  | 8.93 | 28.35 | 986.45 | *Thymus pulegioides*  *Hypericum perforatum*  *Potentilla argentea*  *Lotus cornuculatus*  *Euphorbia cyparissias*  *Trifolium repens* | *Brachypodium pinnatum*  *Fragaria viridis*  *Achillea milefolium*  *Festca rubra*  *Dactylis glomerata* |
| 2011-2015 | 27 | 3.02 | 1.8 | 2.4 | 0.002 | 26.69 | 10.04 | 31.04 | 937.69 | *Thymus pulegioides*  *Lotus cornuculatus*  *Potentilla argentea*  *Hypericum perforatum*  *Potentilla tabernaemontani*  *Plantago lanceolata* | *Brachypodium pinnatum*  *Fragaria viridis*  *Achillea milefolium*  *Festca rubra*  *Dactylis glomerata* |
| **2008-2015** | **27** | **2.7** | **2.3** | **6.8** | **0.002** | **21.11** | **7.44** | **24.62** | **3156.31** | ***Thymus pulegioides***  ***Hypericum perforatum***  ***Potentilla argentea***  ***Trifolium repens***  ***Lotus corniculatus***  ***Trifolium arvense*** | ***Brachypodium pinnatum***  ***Fragaria viridis***  ***Achillea millefolium***  ***Koeleria pyramidata***  ***Poa pratensis***  ***Festuca rubra*** |

**Table S3** Projection matrix of disturbance life cycle

|  |  |  |  | from |  |  |
| --- | --- | --- | --- | --- | --- | --- |
|  |  | Grass | Age 0 | Age 1 | Age 2 | Age 3 |
|  | Grass | 0.997956 | 0 | 0 | 0 | 0.9722 |
|  | Age 0 | 0.002044 | 0.403511 | 0.1021 | 0.022117 | 0.0278 |
| to | Age 1 | 0 | 0.596489 | 0 | 0 | 0 |
|  | Age 2 | 0 | 0 | 0.8979 | 0 | 0 |
|  | Age 3 | 0 | 0 | 0 | 0.977883 | 0 |

**Table S4** Species with highest scores on first axis in Redundancy analysis testing a difference between vegetation on artificial disturbances and control plots first year after disturbance; Life strategies according to (Grime, 1979): C = competitors, S = Stress-tolerators, R = Ruderals

| **Disturbances** | | | **Controls** | | |
| --- | --- | --- | --- | --- | --- |
| **Species** | **Scores** | **Strategy** | **Species** | **Scores** | **Strategy** |
| *Arabidopsis thaliana* | -0.4899 | R | *Brachypodium pinnatum* | 0.6937 | CS |
| *Viola arvensis* | -0.3871 | R | *Fragaria viridis* | 0.6827 | CSR |
| *Hypericum perforatum* | -0.3834 | C | *Achillea millefolium agg.* | 0.4793 | NA |
| *Euphorbia cyparissias* | -0.3486 | CSR | *Festuca rubra* | 0.4285 | C |
| *Rumex acetosella s.lat.* | -0.3456 | CSR | *Arrhenatherum elatius* | 0.4269 | C |
| *Trifolium arvense* | -0.3244 | CSR | *Veronica chamaedrys* | 0.3745 | CSR |
| *Polygonum rurivagum* | -0.2945 | NA | *Galium mollugo* | 0.3618 | C |
| *Potentila argentea* | -0.2736 | CS | *Dactylis glomerata* | 0.34 | C |
| *Myosotis stricta* | -0.2694 | SR | *Festuca rupicola* | 0.3365 | CS |
| *Fallopia convolvolus* | -0.2382 | CR | *Poa pratensis* | 0.2771 | C |
| *Trifolium repens* | -0.2344 | CSR | *Potentilla tabernaemontani* | 0.2393 | CSR |
| *…* |  |  | *…* |  |  |

Grime, J. P. (1979). *Plant Strategies, Vegetation Processes, and Ecosystem Properties*. Chichester: Wiley.

**Fig. S1** One of 30 artificial disturbances (1 m^2^) with the sampled permanent plot (0.5 × 0.5 m)

**Fig. S2** Ordination of vegetation composition of 45 natural disturbances caused by wild boar (circles) and 45 chosen artificial disturbances from the field experiment (triangles). PCO projection using Bray-Curtis distance. (a) Centroids of groups of samples. Filled symbols are used for rooted plots. Empty symbols are used for control undisturbed plots; (b) The same ordination with species. Grey rectangular shows the position of (a) part of the picture.
